# Commentary: Acetaminophen Enhances the Reflective Learning Process

**DOI:** 10.3389/fpsyg.2019.00705

**Published:** 2019-04-02

**Authors:** Jonathon McPhetres

**Affiliations:** Department of Clinical and Social Sciences in Psychology, University of Rochester, Rochester, NY, United States

**Keywords:** acetaminophen, reflective learning, cognitive performance, comment, categorization, COVIS, psychopharmacology

Pearson et al. (2018) very interesting article “Acetaminophen enhances the reflective learning process.” reports an effect of acetaminophen on reflective processes. By claiming to demonstrate that acetaminophen increases reflective processing, this article brings to mind exciting sci-fi-esque musings about “smart pills,” and even more realistic excitement about an applied use of acetaminophen for pilots, surgeons, and students (or perhaps politicians!). This article is promising and investigates important questions about the connection between physical and cognitive processes which entails even more important implications. Unfortunately, I think this article suffers from several flaws and that Pearson et al. have greatly overstated the findings and the implications. I detail five critiques below.

## The Hypotheses are not Statistically Supported

The two hypotheses Pearson et al. test (overall accuracy: *p* = 0.07; trials to criterion: *p* = 0.06) are not below conventional levels of statistical significance (e.g., *p* < 0.05). Recent research has demonstrated that *p*-values close to the arbitrary 0.05 threshold are unlikely to occur repeatedly and, thus, are less likely to replicate (Simonsohn et al., 2014a,b; Benjamin et al., 2018; Camerer et al., 2018). Instead of seeking stronger evidence, the authors go on to examine interactions, and conduct exploratory tests which do not provide much stronger evidence for the claims.

While I commend the authors for clearly distinguishing between exploratory and confirmatory tests, these exploratory tests should be followed up with a clear replication of the effects in an independent dataset (Forstmeier et al., 2017). To be sure, there are ways to overcome unlikely *p*-values and “small” effect sizes—for example, using a well-powered sample, preregistering the initial study, or including a preregistered study directly replicating the effect—but this research does not demonstrate those qualities.

Finally, a particularly astute reviewer also pointed out an additional concern regarding the statistical reporting: the effect sizes are reported incorrectly. Indeed, eta-squared can be calculated from the information reported in the article using the following formula (Cohen, 1965):

$$\begin{array}{l} \eta_{p}^{2}=\;\frac{F\;\times \;df_{effect}}{F\times \;df_{effect}+df_{error}} \end{array}$$

Using this formula, the three eta-squared values reported on page 1,032 (0.02, 0.03, and 0.02) should be 0.04, 0.06, and 0.04, respectively.

## The Follow-Up Tests are Unwarranted

Despite the non-significance of the key interactions, the authors conduct follow-up tests of simple effects which appear to be the tests on which they rest their main claims. This is inappropriate. Further, it appears that the first test on “influence of acetaminophen on reaching criterion” (p. 1,032) showed no significant results, so the authors recoded the criterion variable dichotomously (yes/no) and reconducted the analysis, interpreting it as supporting their hypothesis (*p* = 0.049).

## The Sample Size is Unjustifiably Small

There is no discussion of power or expected effect size. Recommendations have been made to increase sample sizes to at least 100 *per cell* (Simmons et al., 2018) to reduce false positives. Given the recent literature on issues of replicability in the social sciences (e.g., Open Science Collaboration, 2015; Forstmeier et al., 2017; Camerer et al., 2018), the authors should justify such a small sample in a between-subjects design. Better yet, a direct replication of these effects with a larger sample would yield more convincing evidence.

## The Authors Make Use of Many Researcher Degrees of Freedom Which Undermine the Credibility of Their Results

The authors utilize at least 11 researcher degrees of freedom (Wicherts et al., 2016), which are arbitrary decisions made by researchers during various phases of research, all of which increase the likelihood of false positives. These instances are outlined in Table 1. For example, participants were excluded for multiple reasons, including for performance on the main DV. We are left to wonder whether including these participants influences the results in any way. Further, the DVs were recoded in multiple ways, including dichotomizing a non-significant continuous score (trials to criterion) and testing an arbitrary subset of trials (i.e., accuracy prior to the first rule switch), further increasing the likelihood of a false positive result (Simmons et al., 2011).

**Table 1:** Researcher degrees of freedom according to Wicherts et al. (2016).

**Degree of freedom code (Wicherts et al., 2016)**	**Pearson et al. (2018) example**	**Pearson et al. (2018) location**
T2: Vague hypotheses	“We anticipated that acetaminophen would enhance effortful, reflective learning, and decrease reliance on intuitive, reflexive learning strategies” “Enhanced” and “poorer” performance	Page 1,030, last full paragraph Page 1,032, first paragraph
D5: Measuring additional variables	Depression scale Task performance at “chance level” Trials to criterion, learning rate	Page 1,030 Page 1,031 Page 1,032
D6: Lack of power analysis	No justification for sample size given	N/A
D7: No Sampling plan specified	No sampling plan specified	N/A
A1/2: Vague exclusion criteria and “data cleaning”	Low score on depressive symptoms Lacking “complete” task data Task performance “at or below chance”	Page 1,030 Page 1,030 Page 1,031
A3: Treating statistical abnormalities *ad*-*hoc*	Because of possible suppression “… exploratory analysis was conducted to examine accuracy until the first rule change” Recoding trials to criterion as Yes/No “since the majority of participants failed to reach criterion …”	Page 1,032 Page 1,032
A6: Multiple scorings of the DV	Overall accuracy score Accuracy until the first rule change Number of trials to criterion Dichotomizing reaching criterion (Yes/No) on the reflexive-optimal task	Page 1,032 Page 1,032 Page 1,032 Page 1,032
R1: Reproducibility is not assured	Data is not publicly available and not shared	N/A
R5: Misreporting results	Eta-squared values are calculated incorrectly (noticed by a reviewer)	Page 1,032
R6: Presenting exploratory results as confirmatory (HARKing)	Trials to criterion scores Learning rate analysis	Page 1,032 Page 1,032

## The Implications of the Research are Severely Overstated

Given the above limitations, the implications of these findings are greatly overstated. For example, on page 1,033 in the first full paragraph, the authors state “To the degree that people who take acetaminophen are trying to make decisions with clear, logical rules, their performance may improve.”

In the same paragraph, the authors also state “… acetaminophen could potentially help people make difficult decisions by reducing emotional responses to affective contexts while at the same time facilitating more deliberative, effortful information processing …” The present results do not show that acetaminophen facilitates deliberative processing, nor does the study investigate emotions in any way. In the final paragraph on page 1,033, the authors conclude “We found that reflective-optimal decision-making can be enhanced by acetaminophen.”

## Final Comments

In summary, while the results seem exciting at first glance, there are several limitations of these results and the implications are overstated. This is an interesting and very important area of research to be sure, but this is precisely the reason why research on this topic needs to be rigorous and accurate. The average reader or lay-person may read this article and leave believing some effect to be true when, in fact, the article offers very weak evidence for this effect. It would be extremely unfortunate if a person began taking acetaminophen believing it to improve learning. Indeed, as a reviewer pointed out, consumers may reason that, because taking the amount described in the article (1,000 mg) makes one somewhat smarter, then taking 10 times that amount (10 g) may make one ten times as smart. This is a large possible ethical consequence which seems to have not been considered. In short, caution is urged when interpreting these research findings, especially if they are to be used to inform interventions or as a basis for future research.

## Author Contributions

The author confirms being the sole contributor of this work and has approved it for publication.

### Conflict of Interest Statement

The author declares that the research was conducted in the absence of any commercial or financial relationships that could be construed as a potential conflict of interest.
